# To Clear a Room of Mosquitoes

**Published:** 1870-01

**Authors:** 


To Clear a Room of Mosquitoes.—A writer in a South Carolina paper says: "I have tried the following, and find it works like a charm. Take of gum camphor a piece about one-third the size of an egg, and evaporate it by placing it in a tin vessel and hold it over a lamp or candle, taking care it does not ignite. The smoke will soon fill the room and expel the mosquitoes. One night I was terribly annoyed by them, when I thought of and tried the above, after which I never saw nor heard them that night, and the next morning there was not one to be found in the room, though the window had been open all night."—*Druggists' Circular.*

